# Circulating Tissue Inhibitor of Metalloproteinase-4 levels are not a Predictor of Preeclampsia in the period between 20 and 25 Weeks of Gestation

**DOI:** 10.1055/s-0038-1676056

**Published:** 2018-12

**Authors:** Valeria Cristina Sandrim, Jackeline Machado, Heloisa Bettiol, Marco Antonio Barbieri, Viviane Cunha Cardoso, Ana Carolina Palei, Ricardo Carvalho Cavalli

**Affiliations:** 1 Universidade Estadual Paulista Júlio de Mesquita Filho, Botucatu, SP, Brazil; 2 Universidade de São Paulo, Ribeirao Preto, SP, Brazil; 3 University of Mississippi Medical Center, Jackson, MS, United States

**Keywords:** preeclampsia, TIMP-4, TIMPs, MMPs, prediction, circulating biomarkers, angiogenic biomarkers, preeclâmpsia, TIMP-4, TIMPs, MMPs, predição, biomarcadores circulantes, biomarcadores angiogênicos

## Abstract

**Objective** To evaluate whether the circulating level of tissue inhibitor of metalloproteinase-4 (TIMP-4) in the period between 20 and 25 weeks of gestation is a predictor of preeclampsia.

**Methods** We have performed a case-control study, nested in a prospective study cohort in Ribeirão Preto, in the state of São Paulo, Brazil. Of the 1,400 pregnant women evaluated between 20 and 25 weeks of gestation, 460 delivered in hospitals outside of our institution. Of the 940 pregnant women who completed the protocol, 30 developed preeclampsia. Healthy pregnant women (controls, *n* = 90) were randomly selected from the remaining 910 participants. From blood samples collected between 20 and 25 weeks of gestation, we performed a screening of 55 angiogenesis-related proteins in 4 cases and 4 controls. The protein TIMP-4 was the most differentially expressed between cases and controls. Therefore, we measured this protein in all cases (*n* = 30) and controls selected (*n* = 90).

**Results** There were no differences in the plasma TIMP-4 levels of cases compared with controls (1,144 ± 263 versus 1,160 ± 362 pg/mL, respectively; *p *> 0.05).

**Conclusion** Plasma TIMP-4 levels were not altered at 20 to 25 weeks of gestation, before the manifestation of clinical symptoms; therefore, they are not good predictors of the development of preeclampsia.

## Resumo

**Objetivo** Avaliar se o nível de inibidor tecidual de metaloproteinases tipo-4 (TIMP-4, na sigla em inglês) circulante no período entre 20 e 25 semanas de gestação é um preditor de preeclâmpsia.

**Métodos** Foi realizado um estudo caso-controle aninhado em uma coorte de estudo prospectivo em Ribeirão Preto, São Paulo, Brasil. De 1.400 mulheres grávidas avaliadas entre 20 e 25 semanas de gestação, 460 tiveram parto em hospitais fora da nossa instituição. Das 940 gestantes que completaram o protocolo, 30 desenvolveram preeclâmpsia. Gestantes saudáveis (controles, *n* = 90) foram selecionadas aleatoriamente das 910 participantes restantes. A partir de amostras de sangue coletadas entre 20 e 25 semanas de gestação, foi realizada uma triagem de 55 proteínas relacionadas à angiogênese em 4 casos e 4 controles. A proteína TIMP-4 foi a mais diferentemente expressa entre os casos e os controles; portanto, medimos esta proteína em todos os casos (*n* = 30) e controles selecionados (*n* = 90).

**Resultados** Não houve diferenças nos níveis plasmáticos de TIMP-4 nos casos em comparação com os controles (1.144 ± 263 versus 1.160 ± 362 pg/mL, respectivamente; *p *> 0,05).

**Conclusão** Os níveis plasmáticos de TIMP-4 não foram alterados no período entre 20 e 25 semanas de gestação antes da manifestação dos sintomas clínicos; portanto, não são um bom preditor do desenvolvimento da preeclâmpsia.

## Introduction

The early identification of pregnant women at high risk of developing preeclampsia is important, allowing the tracking and reduction of complications for the mother and the fetus. In recent years, several circulating biomarkers have been explored, such as antiangiogenic molecules, homocysteine, oxidative stress markers and vasoactive peptides.^1^ ^2^ ^3^ ^4^ The most promising biomarker is the combination of increased levels of the antiangiogenic factor soluble fms-like tyrosine kinase-1 (sFLT-1) concurrent with decreased placental growth factor (PIGF).^5^ Soluble fms-like tyrosine kinase-1, is a splicing variant of fms related tyrosine kinase (FLT-1) (one of the receptors of vascular endothelial growth factor [VEGF]), and it contributes to preeclampsia pathogenesis by antagonizing VEGF and PIGF receptor binding, thus reducing their biological action on angiogenesis and endothelial function.^6^

Although it is currently widely accepted that an antiangiogenic profile contributes to the pathophysiology of preeclampsia, few angiogenesis-based biomarkers have been explored in prediction studies, which include measurements of endostatin and of angiopoetin-1/2.^7^ ^8^ ^9^

In the present study, we first screened 55 proteins related to angiogenesis in the plasma of pregnant women who subsequently developed preeclampsia (cases, *n* = 4) compared with pregnant women who were healthy until delivery (control, *n* = 4), collected at between 20 and 25 weeks of gestation. The most expressed protein in this case was the tissue inhibitor of metalloproteinase-4 (TIMP-4). Therefore, our next step was to validate the findings of the screening in a larger number of pregnant women (30 cases and 90 controls).

## Methods

### Study Design and Population

The present nested cohort study is part of a broader observational prospective study whose main aims were to assess new risk factors for preterm birth and the impact of perinatal indicators in fetal and infant growth in two different Brazilian cohorts: one in the city of Ribeirão Preto, in the state of São Paulo, Brazil, and the other in the city of São Luis, Maranhão, Brazil (the BRISA cohort).^10^ The data reported here is only related to the cohort of Ribeirão Preto. Pregnant women were recruited at hospitals and healthcare units during their 1^st^ trimester prenatal visit and were invited to go to the Hospital das Clínicas da Faculdade de Medicina de Ribeirão Preto, Universidade de São Paulo (HCFMRP-USP, in the Portuguese acronym) to participate in the study.

**Fig. 1:**
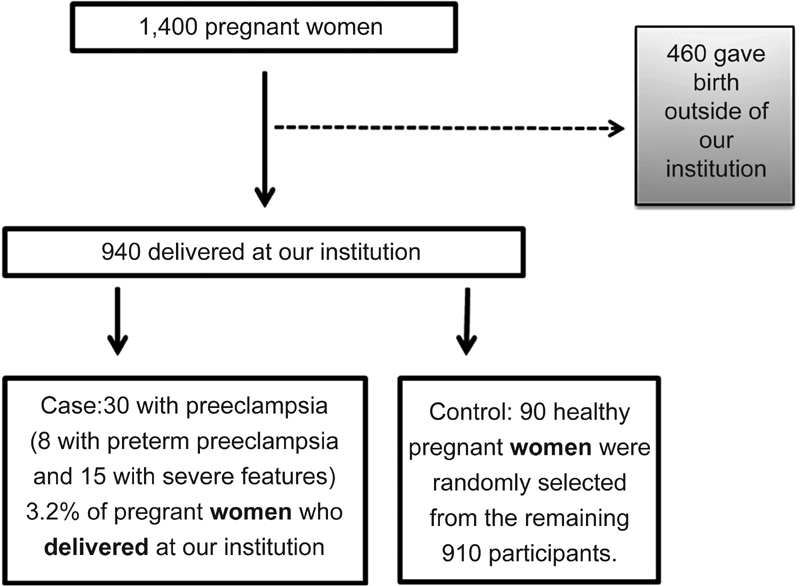
Flowchart of the participant enrollment.

The participants enrolled in the study were recruited as follows (Fig. 1): out of 1,400 pregnant women evaluated at the HCFMRP-USP in the period between 20 and 25 weeks of gestation, 460 had deliveries in hospitals outside of our institution. Out of 940 pregnant women who completed the protocol in our institution, 30 developed preeclampsia later (cases). Healthy pregnant women (controls, *n* = 90) were randomly selected from the remaining 910 participants. The present study was approved by the Institutional Review Board (#4116/2008). All of the participants provided a written informed consent. All of the study procedures comply with the principles of the Declaration of Helsinki.

Preeclampsia was defined according to the American College of Obstetricians and Gynecologists (ACOG).^11^ At the time of clinic attendance, during the 2^nd^ trimester of gestation (at 20–25 weeks), maternal venous blood samples were collected into Vacutainer K_2_EDTA tubes (BD, Franklin Lakes, NJ, USA) and immediately centrifuged. Plasma samples were stored at - 80° C until used to measure biomarkers.

#### Human Angiogenesis Array Kit and Enzyme-Linked Immunosorbent Assay (ELISA)

First, we randomly selected 4 cases and 4 controls from the sample population to perform a screening of 55 angiogenesis-related proteins in plasma samples collected in the period between 20 and 25 weeks of gestation. Simultaneous screening of the relative levels of 55 angiogenesis-related proteins was performed using the ARY007 Proteome Profiler Human Angiogenesis Array Kit (R&D Systems, Minneapolis, MN, USA), according to the protocol of the manufacturer. Expression profiles were generated by quantifying the mean pixel densities from the array membranes with the ImageJ software 1.8.0 (U. S. National Institutes of Health, Bethesda, MD, USA).

**Fig. 2:**
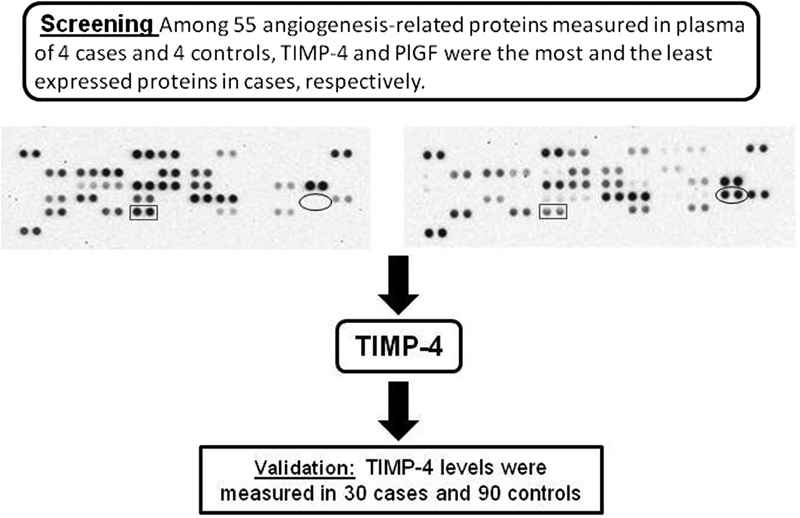
Schematic diagram depicting the screening and validation steps focused on the investigation of angiogenic proteins that could be used as a predictor biomarker of preeclampsia in the period between 20 and 25 weeks of gestation. Tissue inhibitor of matrix metalloproteinase-4 (TIMP-4) and placental growth factor (PlGF) coordinates represented by oval and rectangle formats, respectively, in representative protein array blots.

Of all the proteins evaluated, TIMP-4 was the one that presented the highest levels in case samples compared with controls. To validate this finding, we measured the plasma TIMP-4 concentration in all cases (*n* = 30) and in all controls (*n* = 90) by ELISA using the DTM400 Quantikine kit (R&D Systems, Minneapolis, MN, USA), following the protocol of the manufacturer (Fig. 2).

### Statistical Analysis

Graphs and statistical analyses were generated with the GraphPad Prism 6.0 software (GraphPad Software, La Jolla, CA, USA). The clinical characteristics and TIMP-4 levels of the studied groups were compared by using the Student *t*-test or the chi-squared (χ^2^) test. A probability value of *p* < 0.05 was considered the level of statistical significance.

### Ethical Approval and Consent of Participants

The present study was approved by the Institutional Review Board of the HCFMRP-USP (reference 4116/2008, approval date: November 11, 2008). All of the participants provided written informed consent.

## Results

At first, we performed a screening of 55 angiogenesis-related proteins in plasma collected in the period between 20 and 25 weeks of gestation from pregnant women who subsequently developed preeclampsia (cases, *n* = 4) and from pregnant women who remained healthy until delivery (controls, *n* = 4). All the proteins evaluated in controls and cases are shown in Table S1. From 55 proteins, 27 and 24 were not detected in cases and controls, respectively (Table S1). The proteins that have reached at least 30% of differential expression between cases and controls were: heparin-binding epidermal growth factor (HB-EGF), metalloproteinase-9 (MMP-9), TIMP-4, and PIGF. We decided to focus the subsequent experiments on TIMP-4 because, first, PIGF has been extensively explored in preeclampsia (and our data is in line with these studies, that is, reduced levels in pregnant women who developed preeclampsia later); secondly, HB-EGF was not detected in 2 samples of each group; and third, the variability of MMP-9 levels was extremely high among the samples.

**Table 1:** Characteristics of pregnant women enrolled in the period between 20 and 25 weeks of gestation

Parameters	Control	Case
*n*	90	30
Age (years old)	26 ± 6	28 ± 7
Primiparas (%)	35	36
GA Sampling (weeks)	23 ± 1	23 ± 1
Heart rate (bpm)	80 ± 10	79 ± 10
SBP (mm Hg) at sampling	108 ± 12	115 ± 13*
DBP (mm Hg) at sampling	66 ± 7	74 ± 8*
Newborn weight (g)	3,336 ± 456	2,839 ± 1,002*
APGAR Score 1 (1 minute) < 7	15	4
APGAR Score 2 (5 minute) < 7	1	1
Preterm preeclampsia (%)		27
Cases with severe features (%)		50

Abbreviations: bpm, beats per minute; DBP, diastolic blood pressure; GA, gestational age; NBW, newborn weight; SBP, systolic blood pressure.

Information in this table refers to the moment of blood collection before the diagnosis of preeclampsia, except for NBW. Data is represented as mean ± standard deviation (SD) or n (percentage of total).

* *vs* control (healthy pregnant during the whole gestation).

To validate these findings, we measured the plasma TIMP-4 concentration in controls (*n* = 90) and in cases (*n* = 30). The clinical characteristics of pregnant women at enrollment for these two study groups are shown in Table 1. Even before the clinical onset of preeclampsia, systolic blood pressure (SBP) and diastolic blood pressure (DBP) levels were significantly higher in cases compared with controls (*p* = 0*.*015 and *p* < 0.0001, respectively, Table 1). The gestational age was similar between the groups (*p* > 0.05, Table 1), which is particularly relevant when evaluating circulating biomarkers.

**Fig. 3:**
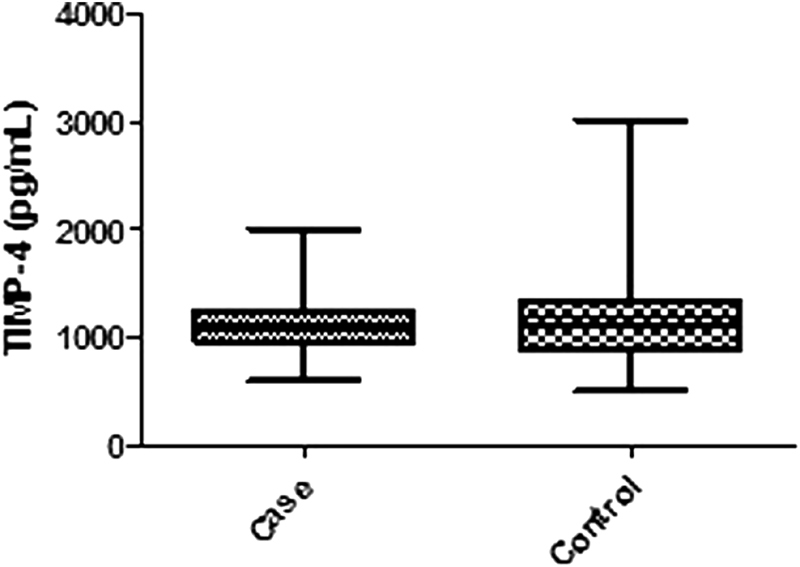
Validation step measuring plasma tissue inhibitor of matrix metalloproteinase-4 (TIMP-4) concentration in cases and controls. Boxes extend from the 25th percentile to the 75th percentile, with a line at the median. Whiskers show the highest and the lowest values. *P-value* = 0.79, using the Mann-Whitney test.

Intriguingly, ELISA did not confirm the results obtained with the human angiogenesis array, and there were no differences in the plasma TIMP-4 levels of cases compared with controls (1,144 ± 263 versus 1,160 ± 362 pg/mL, respectively; *p *>* 0.05*, Fig. 3). Moreover, the plasma TIMP-4 levels were similar between preterm and term preeclampsia women (1,014 ± 221 pg/mL versus 1,202 ± 268 pg/mL, respectively, *p *> 0.05).

## Discussion

This is the first study investigating circulating TIMP-4 levels as a predictor of development of preeclampsia; we found that this protein is not a useful biomarker in the period between 20 and 25 weeks of gestation. Although our screening using a protein array suggested that TIMP-4 levels are increased in the circulation of pregnant women who subsequently develop preeclampsia, we were not able to confirm this result when the plasma TIMP-4 concentration was determined in a larger sample size using ELISA. Recently, our group demonstrated that women with preeclampsia (after the development of the syndrome) present higher levels of plasma TIMP-4, showing the impact of this protein when the syndrome is established.^12^

Tissue inhibitors of metalloproteinases (TIMPs) are a family of proteins that bind to the active site of matrix metalloproteinases (MMPs), endogenously inhibiting their enzymatic activity.^13^ Although the four known TIMPs (1, 2, 3 and 4) block MMP-mediated proteolysis of extracellular matrix components, they show several regulatory, structural, biochemical, and tissue expression differences, as well as an MMP-independent role in cell signaling.^13^ ^14^ The expression of TIMP-4 is very selective, being expressed predominately in the heart and in the brain. Increased TIMP-4 levels have been found in atherosclerosis, myocardial infarction, left ventricular remodeling, and diastolic heart failure.^15^ ^16^

Recently, Zhang et al^17^ demonstrated that increased maternal plasma TIMP-4 levels in the period between 8 and 20 weeks of gestation may serve as a predictive biomarker for pregnancy-induced hypertension (∼ 22% higher), which is defined as the development of hypertension during pregnancy in a previously normotensive woman, as demonstrated by proteinuria or more than 2 measurements of SBP ≥140 mm Hg or DBP ≥90 mm Hg taken at least 4 hours apart.^17^ Our results do not support this finding, probably because we measured the levels of TIMP-4 in the second trimester of pregnancy (20–25 weeks) and Zhang et al^17^ measured them between 8 and 20 weeks. Moreover, these authors did not differentiate pregnancy-induced hypertension from preeclampsia and other related hypertensive complications.

Part of the biological function of TIMP-4 is due to its inhibition of some MMPs, such as MMP-1 (IC_50_ of 19 nM), MMP-2 (IC_50_ of 3 nM), MMP-7 (IC_50_ of 8 nM), and MMP-9 (IC_50_ of 83 nM).^18^ Matrix metalloproteinases actively participate in placentation, as well as in trophoblast invasion and in endothelial cell differentiation of the uterine spiral arteries.^19^ Therefore, it is possible that Zhang et al^17^ have found increased levels of TIMP-4 in the period between 8 and 20 weeks of gestation because, during this gestational period, TIMP-4 is more important for placentation-related processes, compared with the period between 20 and 25 weeks, which was used in our study. Further studies are needed to assess circulating and placental TIMP-4 expression longitudinally, in low- and high-risk pregnancies, to determine the role of TIMP-4 in the pathophysiology of preeclampsia and to confirm the applicability of this angiogenic protein as a predictive biomarker.

The limitations of our study include: 1) the measurement of TIMP-4 uses plasma, and this tissue probably represents the sum of all TIMP-4 production in body tissues; and 2) TIMP-4 was not measured before 20 weeks of gestation, which may be more relevant to the role of TIMP-4 in preeclampsia.

## Conclusion

Plasma TIMP-4 levels were not altered in the period between 20 and 25 weeks of gestation, before the manifestation of the clinical symptoms of preeclampsia. Therefore, other non-invasive biomarkers of the second trimester of pregnancy should be developed to improve the maternal and fetal outcomes, mainly in developing countries.
