# Supplementary material for: Circulating Tissue Inhibitor of Metalloproteinase-4 levels are not a Predictor of Preeclampsia in the period between 20 and 25 Weeks of Gestation
Source: Rev Bras Ginecol Obstet. 2018 Dec;40(12):757–62. doi: 10.1055/s-0038-1676056 (PMC10316924; doi:10.1055/s-0038-1676056)
Supplement: Supplementary file 1 — Supplementary Material [file 10-1055-s-0038-1676056-s180218.pdf]

**Table S1** Human angiogenesis array coordinates

| | 1 | 2 | 3 | 4 | 5 | 6 | 7 | 8 | 9 | 10 | 11 | 12 | 13 | 14 | 15 | 16 | 17 | 18 | 19 | 20 | 21 | 22 | 23 | 24 |
|---|---|---|---|---|---|---|---|---|---|---|---|---|---|---|---|---|---|---|---|---|---|---|---|---|
| A | o | o | | | o | o | o | o | o | o | o | o | o | o | o | o | o | o | o | o | | | o | o |
| B | o | o | o | o | o | o | o | o | o | o | o | o | o | o | o | o | o | o | o | o | o | o | o | o |
| C | o | o | o | o | o | o | o | o | o | o | o | o | o | o | o | o | o | o | o | o | o | o | o | o |
| D | o | o | o | o | o | o | o | o | o | o | o | o | o | o | o | o | o | o | o | o | o | o | o | o |
| E | o | o | o | o | o | o | o | o | o | o | o | o | o | o | o | o | o | o | o | o | o | o | | |
| F | o | o | | | | | | | | | | | | | | | | | | | | | o | o |

| | | Mean pixel density | | Case/Control |
|---|---|---|---|---|
| | | Control | Case | Ratio |
| Activin A | A5, A6 | – | – | – |
| ADAMTS-1 | A7, A8 | – | – | – |
| Amphiregulin (AR) | A17, A18 | – | – | – |
| Angiogenin (ANG) | A9, A10 | 37,087 | 34,919 | 0.94 |
| Angiopoietin-1 (Ang-1) | A11, A12 | 20,518 | 25,228 | 1.23 |
| Angiopoietin-2 (Ang-2) | A13, A14 | – | – | – |
| Angiostatin/Plasminogen | A15, A16 | 12,720 | 15,131 | 1.19 |
| Artemin | A19, A20 | – | – | – |
| Coagulation Factor III (TF) | B1, B2 | – | – | – |
| CXCL16 | B3, B4 | 23,799 | 20,665 | 0.87 |
| DPPIV (CD26) | B5, B6 | 26,237 | 21,358 | 0.81 |
| EGF | B7, B8 | 15,397 | 19,511 | 1.27 |
| PK1 | B9, B10 | 7,135 | – | – |
| Endoglin (CD105) | B11, B12 | 29,162 | 28,257 | 0.97 |
| Endostatin/Collagen XVIII | B13, B14 | 23,771 | 21,159 | 0.89 |
| Endothelin-1 (ET-1) | B15, B16 | – | – | – |
| FGF acidic (FGF-1) | B17, B18 | 8,764 | – | – |
| FGF basic (FGF-2) | B19, B20 | – | – | – |
| FGF-4 | B21, B22 | – | – | – |
| FGF-7 (KGF) | B23, B24 | – | – | – |
| GDNF | C1, C2 | – | – | – |
| GM-CSF | C3, C4 | – | – | – |
| HB-EGF | C5, C6 | 7,348 | 13,016 | **1.77** |
| HGF | C7, C8 | 8,278 | 10,016 | 1.21 |
| IGFBP-1 | C9, C10 | 36,753 | 32,459 | 0.88 |
| IGFBP-2 | C11, C12 | 32,815 | 35,191 | 1.07 |
| IGFBP-3 | C13, C14 | 29,972 | 25,479 | 0.85 |
| IL-1β (IL-1F2) | C15, C16 | 5,439 | – | – |
| IL-8 (CXCL8) | C17, C18 | – | – | – |
| LAP (TGF-β1) | C19, C20 | 8,906 | 8,998 | 1.01 |
| Leptin | C21, C22 | 47,311 | 41,011 | 0.87 |
| MCP-1 (CCL2) | C23,C24 | – | – | – |
| MIP-1α (CCL3) | D1, D2 | – | – | – |
| MMP-8 | D3, D4 | 13,772 | 10,132 | 0.74 |
| MMP-9 | D5, D6 | 18,876 | 26,262 | **1.39** |

(*Continued*)

**Table S1** (*Continued*)

| | 1 | 2 | 3 | 4 | 5 | 6 | 7 | 8 | 9 | 10 | 11 | 12 | 13 | 14 | 15 | 16 | 17 | 18 | 19 | 20 | 21 | 22 | 23 | 24 |
|---|---|---|---|---|---|---|---|---|---|---|---|---|---|---|---|---|---|---|---|---|---|---|---|---|
| NRG1-β1 (HRG1-β1) | | | | | | | | D7,D8 | | | | – | | | – | | | – | | | | | | |
| PD-ECGF | | | | | | | | D11, D12 | | | | – | | | – | | | – | | | | | | |
| PDGF-AA | | | | | | | | D13, D14 | | | | 25,841 | | | 32,287 | | | 1.25 | | | | | | |
| PDGF-AB/PDGF-BB | | | | | | | | D15, D16 | | | | 34,454 | | | 32,427 | | | 0.94 | | | | | | |
| Pentraxin 3 (PTX3) | | | | | | | | D9, D10 | | | | 15,364 | | | 13,560 | | | 0.88 | | | | | | |
| Persephin | | | | | | | | D17, D18 | | | | – | | | – | | | – | | | | | | |
| Platelet Factor 4 (PF4) | | | | | | | | D19, D20 | | | | 3,798 | | | 5,019 | | | **1.32** | | | | | | |
| PlGF | | | | | | | | D21, D22 | | | | 16,880 | | | 6,233 | | | **0.37** | | | | | | |
| Prolactin | | | | | | | | D23, D24 | | | | 22,129 | | | 16,314 | | | 0.74 | | | | | | |
| Serpin B5 (Maspin) | | | | | | | | E1, E2 | | | | – | | | – | | | – | | | | | | |
| Serpin E1 (PAI-1) | | | | | | | | E3, E4 | | | | 34,264 | | | 29,026 | | | 0.85 | | | | | | |
| Serpin F1 (PEDF) | | | | | | | | E5, E6 | | | | – | | | – | | | – | | | | | | |
| Thrombospondin-1 (TSP-1) | | | | | | | | E11, E12 | | | | – | | | – | | | – | | | | | | |
| Thrombospondin-2 (TSP-2) | | | | | | | | E13, E14 | | | | – | | | – | | | – | | | | | | |
| TIMP-1 | | | | | | | | E7, E8 | | | | 24,242 | | | 24,889 | | | 1.03 | | | | | | |
| TIMP-4 | | | | | | | | E9, E10 | | | | 27,007 | | | 35,826 | | | **1.33** | | | | | | |
| uPA | | | | | | | | E15, E16 | | | | 15,479 | | | 11,401 | | | 0.74 | | | | | | |
| Vasohibin | | | | | | | | E17,E18 | | | | – | | | – | | | – | | | | | | |
| VEGF-A | | | | | | | | E19,E20 | | | | 11,769 | | | 10,895 | | | 0.93 | | | | | | |
| VEGF-C | | | | | | | | E21,E22 | | | | – | | | – | | | – | | | | | | |
| Negative Control | | | | | | | | F23,F24 | | | | – | | | – | | | – | | | | | | |

Abbreviations: ADAMTS, A disintegrin-like and metalloproteinase with thrombospondin; CCL2, chemokine ligand-2; CCL3, chemokine ligand-3; CXCL8, chemokine ligand-8; CXCL16, chemokine ligand 16; DPPIV, dipeptidyl peptidase IV; EGF, epidermal growth factor, FGF, fibroblast growth factor; GDNF, glial-cell line-derived neurotrophic factor; GM-CSF, granulocyte macrophage colony stimulating factor; HB-EGF, heparin binding epidermal growth factor, HGF, hepatocyte growth factor; HRG1-β1, heregulin1-Beta1; IGFBP-1, insulin-like growth factor binding protein-1; IGFBP-2, insulin-like growth factor binding protein-2; IGFBP-3, insulin-like growth factor binding protein-3; IL-1β,, interleukin 1-Beta; IL-8, interleukin-8; KGF, keratinocyte growth factor; LAP, leukemia associated protein; MCP-1, monocyte chemoattractant protein-1; MIP-1α, macrophage inflammatory protein-1 Alpha; MMP-8, matrix metalloproteinase-8; MMP-9, matrix metalloproteinase-9; NRG1-β1, neuregulin-1 Beta-1; PD-ECGF, Platelet-derived endothelial cell growth factor; PDGF-AA, platelet-derived growth factor-AA; PDGF-AB, platelet-derived growth factor-AB; PDGF-BB, platelet-derived growth-factor-BB; PK1, pyruvate kinase1; PlGF, placental growth factor; TGF-β1, transforming growth factor Beta-1; TIMP-1, tissue inhibitor of metalloproteinase-1; TIMP-4, tissue inhibitor of metalloproteinase-4; uPA, urokinase-like plasminogen activator; VEGF-A, vascular endothelial cell growth factor-A; VEGF-C, vascular endothelial growth factor-C.
**Bold** – proteins with 30% difference between cases and controls.
